# Exopolysaccharides From *Lactobacillus paracasei* Isolated From Kefir as Potential Bioactive Compounds for Microbiota Modulation

**DOI:** 10.3389/fmicb.2020.583254

**Published:** 2020-10-16

**Authors:** Ana Agustina Bengoa, Carolina Dardis, Nina Gagliarini, Graciela L. Garrote, Analía G. Abraham

**Affiliations:** ^1^Facultad de Ciencias Exactas, Centro de Investigación y Desarrollo en Criotecnología de Alimentos, Universidad Nacional de La Plata – Consejo Nacional de Investigaciones Científicas y Técnicas Centro Científico-Tecnológico La Plata – Comisión de Investigaciones Científicas de la Provincia de Buenos Aires, La Plata, Argentina; ^2^Área Bioquímica y Control de Alimentos – Facultad de Ciencias Exactas, Universidad Nacional de La Plata, La Plata, Argentina

**Keywords:** prebiotics, probiotics, microbiota, short chain fatty acids, exopolysaccharide, lactic acid bacteria

## Abstract

Microbiota coexists in true symbiosis with the host playing pivotal roles as a key element for well-being and health. Exopolysaccharides from lactic acid bacteria are an alternative as novel potential prebiotics that increase microbiota diversity. Considering this, the aim of the present work was to evaluate the capacity of the EPS produced by two *L. paracasei* strains isolated from kefir grains, to be metabolized *in vitro* by fecal microbiota producing short chain fatty acids. For this purpose, fecal samples from healthy children were inoculated in a basal medium with EPS and incubated in anaerobiosis at 37°C for 24, 48, and 72 h. DGGE profiles and the production of SCFA after fermentation were analyzed. Additionally, three selected samples were sequenced by mass sequencing analysis using Ion Torrent PGM. EPS produced by *L. paracasei* CIDCA 8339 (EPS_8339_) and CIDCA 83124 (EPS_83124_) are metabolized by fecal microbiota producing a significant increase in SCFA. EPS_8339_ fermentation led to an increment of propionate and butyrate, while fermentation of EPS_83124_ increased butyrate levels. Both EPS led to a profile of SCFA different from the ones obtained with inulin or glucose fermentation. DGGE profiles of 72 h fermentation demonstrated that both EPS showed a different band profile when compared to the controls; EPS profiles grouped in a cluster that have only 65% similarity with glucose or inulin profiles. Mass sequencing analysis demonstrated that the fermentation of EPS_8339_ leads to an increase in the proportion of the genera *Victivallis*, *Acidaminococcus* and *Comamonas* and a significant drop in the proportion of enterobacteria. In the same direction, the fermentation of EPS_83124_ also resulted in a marked reduction of Enterobacteriaceae with a significant increase in the genus *Comamonas*. It was observed that the changes in fecal microbiota and SCFA profile exerted by both polymers are different probably due to differences in their structural characteristics. It can be concluded that EPS synthesized by both *L. paracasei* strains, could be potentially used as bioactive compound that modify the microbiota increasing the production of propionic and butyric acid, two metabolites highly associated with beneficial effects both at the gastrointestinal and extra-intestinal level.

## Introduction

Microbiota coexists in true symbiosis with the host playing pivotal roles as a key element for well-being and health (Weiss and Hennet, 2017; Cani, 2018). The relevance of microbial ecology at the intestinal level on health status led the “International Scientific Association for Probiotics and Prebiotics” (ISAPP) to propose the concept of “normobiosis” to characterize a healthy microbiota where microorganisms with potential benefits for health predominate in number compared to potentially harmful ones, in contrast to “dysbiosis” in which one or a few potentially harmful microorganisms are dominant creating a disease situation (Roberfroid et al., 2010; Thursby and Juge, 2017). The intestinal microbiota responds to multiple stress factors such as diet, antibiotic use, inflammation of the intestinal tract and/or infection of the host with enteric pathogens (Conlon and Bird, 2015; Shen and Wong, 2016; Durack and Lynch, 2019). A stable microbiota with an adequate balance is necessary to maintain the integrity of the epithelial barrier, the immune balance and the physiological control of inflammatory processes. In turn, a “dysbiotic” microbial composition leads to an intestine with loss of integrity of the epithelial barrier which favors bacterial translocation and inflammation (Round and Mazmanian, 2009; Tsai et al., 2019). In this context, the use of diet as a basis for modifying the microbiota has re-emerged in recent years, validating the ancient concepts of the relevant role of nutrition in health (Requena et al., 2018). Therefore, the interest for probiotics or novel sources of prebiotic compounds is increasing all over the world (Ewaschuk and Dieleman, 2006; Gareau et al., 2010; Alagón Fernández del Campo et al., 2019; Venema et al., 2020).

Prebiotics has recently been defined as “a substrate that is selectively utilized by host microorganisms conferring a health benefit,” expanding the concept of prebiotics to include non-carbohydrate substances with healthy effects even at distal sites (Gibson et al., 2017). Prebiotics has been studied for the modulation of infant microbiota on account of their long-lasting effects, extended even after the administration period. Otherwise, they have low risk of serious adverse effects and are easy to administrate in infant foods (Miqdady et al., 2020). The administration of prebiotics in children is associated with a number of beneficial health outcomes, such as reduced risk of some allergic reactions, reduced inflammation and risk of infections (Miqdady et al., 2020). Additionally, they may contribute to reduce the risk of development of dysbiosis associated chronic diseases like intestinal bowel disease, irritable bowel syndrome, and type 1 diabetes (Milani et al., 2017). Oligosaccharides present in human milk (HMO) as well as galactooligosacharides (GOS) and fructooligosaccharides (FOS) usually included in infant milk formulae are the most studied prebiotics used for children, which have been proved to induce specific changes in the composition and metabolic activity of the intestinal microbiota (Braegger et al., 2011; Parker et al., 2020). The end products of prebiotics fermentation are acetic, propionic and butyric acids, lactic acid, hydrogen, methane and carbon dioxide (Louis et al., 2014). Lactate and short chain fatty acids (SCFA) are used by host cells as an energy source. They participate as mediators of the host response since they are able to interact with G protein-coupled receptors (GPR43, GPR41, GPR81, and GPR109A) modulating positively or negatively the activity of enzymes that originate second messengers. Besides, some SCFA act as epigenetic regulators by inhibition of histone deacetylases (HDAC) (Kasubuchi et al., 2015). In addition, they can promote the integrity of the epithelial barrier function by reinforcing tight junctions (Morrison and Preston, 2016) and play a role in the regulation of inflammatory response mediated by inflammasome (Offermanns, 2014). They participate in the absorption of water and electrolytes (Vinolo et al., 2011; Koh et al., 2016) and are relevant not only in the context of gastrointestinal pathologies (Louis and Flint, 2009, 2017; Thorburn et al., 2014) but also of extraintestinal diseases (Durack and Lynch, 2019).

Fermented foods containing lactic acid bacteria, whether probiotic or not, are the main source of microorganisms that temporarily complete the microbial community of the gastrointestinal tract, constituting what is known as the transient microbiome (Koh et al., 2016) that can reach 10^10^–10^11^ viable bacteria ingested per day, depending on the eating habits of each individual (Plé et al., 2015; Rezac et al., 2018). Some lactic acid bacteria produce exopolysaccharides (EPS) during fermentation, which, when ingested with the fermented product, can serve as a substrate for commensal bacteria (Ryan et al., 2015), stimulating the development of beneficial microorganisms at the intestinal level and the production of bioactive metabolites (Salazar et al., 2011; Hamet et al., 2016). Being selectively fermented by the microbiota, exopolysaccharides from lactic acid bacteria constitute an alternative as novel potential prebiotic compounds (Balzaretti et al., 2017; Lynch et al., 2018).

Kefir is an artisanal fermented food obtained by milk fermentation with the complex microbiota present in kefir grains. This fermented milk has a long tradition of offering health benefits such as antimicrobial activity, stimulation of immune system, anti-inflammatory, anti-obesity, cholesterol lowering and antioxidant effects, improvement of lactose tolerance, and enhancement of intestinal bacterial microbiota, among others. Lactic acid bacteria, yeast and acetic acid bacteria of different genera, species and even strains coexist in this product and they and/or the metabolites synthesized by them during fermentation could contribute to beneficial health properties attributed to its consumption (Garrote et al., 2010; Bengoa et al., 2019b). *Lactobacillus paracasei* CIDCA 8339 and CIDCA 83124 are EPS-producing strains isolated from Argentine kefir grains (Hamet et al., 2013; Bengoa et al., 2018a) that have good technological properties and fulfill safe requirement for food application of Argentine and European regulation (Bengoa et al., 2019a). *L. paracasei* strains produce EPS both in milk (Hamet et al., 2015) and culture media (Bengoa et al., 2018a). Additionally, it has been demonstrated that these strains present potential probiotic properties like the adhesion ability to intestinal epithelial cells which is increased after passage through the gastrointestinal tract (Bengoa et al., 2018b) and the protective effect against *Salmonella* infection *in vitro* (Zavala et al., 2016).

Considering that microorganisms’ metabolites may contribute to health properties of the fermented product, the aim of the present work was to evaluate the capacity of the EPS produced by *L. paracasei* CIDCA 8339 and CIDCA 83124 in milk to be metabolized *in vitro* by fecal microbiota producing short chain fatty acids.

## Materials and Methods

### Microorganisms, Growth Conditions, and Fermented Milks Production

*L. paracasei* CIDCA 8339 and CIDCA 83124 were grown in MRS broth (Difco Laboratories, Detroit, MI, United States) under aerobic conditions at 30°C for 24 h. For fermented milks production, 10 mL of an active culture of the corresponding *L. paracasei* strain containing ≈ 1 × 10^9^ CFU/mL were inoculated in 1,000 mL of UHT low-fat milk (La Serenísima, Mastellone Hnos S.A, Argentina) and then incubated in aerobic conditions at 30°C for 24 h.

### Exopolysaccharide Obtainment

EPS extraction from the fermented milk was performed according to Rimada and Abraham (2003). Fermented milks (500 mL) were heated for 30 min at 100°C to promote the detachment and dissolution of the polysaccharide bound to the cells and the inactivation of enzymes that could hydrolyze EPS. Trichloroacetic acid 8% (Ciccarelli, Santa Fe, Argentina) was added to precipitate proteins and the samples were then centrifuged at 10,000 × g for 20 min at 20°C in an Avanti J25 centrifuge (Beckman Coulter Inc., Carlsbad, CA, United States). The EPS suspended in the supernatant was precipitated by adding two volumes of ethanol per volume of supernatant. Finally, the samples were dialyzed for 48 h at 4°C with stirring through a 1 kDa cut-off dialysis membrane (Spectra/Por, Spectrum laboratories, CA, United States) to remove lactose residues. In order to evaluate samples purity, the protein content was determined qualitatively by the Bradford method (Bradford, 1976). Thin layer chromatography (TLC) was used to determine the absence of lactose and other simple sugars in the EPS samples. EPS were finally lyophilized and preserved at room temperature until use. EPS extraction was performed from two independent cultures.

### EPS Molecular Mass Determination

Average molecular weight (Mw) was determined by high-performance size exclusion chromatography using a OH-PAK SB-805HQ gel filtration column (SHODEX, Kawasaki, Japan) with refractive index (RI) detection system according to Piermaria et al. (2008). Samples were filtered through a 0.45 μm membrane (Millipore Corporation, Milford, MA, United States) and 50 μL of polysaccharide solutions (0.5 g/L in NaNO_3_ 0.1 M) were injected for each run. Samples elution was performed at room temperature using NaNO_3_ 0.1 M as mobile phase with a flow rate of 0.95 mL/min (pressure 120–130 psi). Dextrans with Mw ranging from 97,000 to 3,800,000 Da (ALO-2770, Phenomenex, Torrance, CA, United States) were used as standard.

### *In vitro* EPS Fermentation by Human Fecal Microbiota

Fermentation assay was carried out using fecal samples from five healthy children aged between 8 months and 3 years old. The donors were selected taking into account that they had an optimal health state (normal anthropometric values, without overweight, and without previous pathologies), an omnivorous diet and that they had not consumed antibiotics in the last 6 months prior to the assay. In addition, a survey was conducted to the parents in relation to the aforementioned aspects (Supplementary Figure S1). Samples were collected by the parents according to the protocol and with the sterile materials provided and sent to the laboratory the same day, together with a note of informed consent in obedience to the protocol approved by the Central Bioethical Committee, National University of La Plata (May 2017). All of them were kept at 4°C and processed within 24 h after deposition to guarantee the viability of the microorganisms present.

For the fecal homogenate, equal amounts of the five samples (5 g) were suspended into sterile phosphate buffer saline (225 mL) and mixed to obtain a 1/10 diluted pool (Aguirre et al., 2014). Homogenates were inoculated to a carbohydrate-free basal medium (1/10) with the EPS under study as the only sugar source at a final concentration of 0.3% w/v. The carbohydrate-free basal medium was formulated according to Salazar et al. (2008): peptone water 2 g/L, yeast extract 2 g/L, NaCl 0.1 g/L, K_2_HPO_4_ 0.04 g/L, KH_2_PO_4_ 0.04 g/L, MgSO_4_ 0.01 g/L, CaCl_2_ 6H_2_O 0.01 g/L, NaHCO_3_ 2 g/L, cysteine HCl 2.5 g/L, bile salts 0.5 g/L, and tween 80 2 g/L. The medium was autoclaved and then 1 mL/L of hemin solution (50 mg/ml) and 10 μL/L of vitamin K previously sterilized by filtration were added.

Samples were incubated for 24, 48, or 72 h at 37°C in anaerobiosis using jars (AnaeroPack, Mitsubishi Gas Chemical Company, Japan) according to Salazar et al. (2008). Controls without sugar (BM), with glucose (GLU) (Britania, Buenos Aires, Argentina) and with inulin (INU) (Saporiti, Buenos Aires, Argentina) in the same concentration (0.3% w/v) were included in the experiment. Each fermentation condition was performed in triplicate. After fermentation, the samples were centrifuged for 10 min at 10,000 xg, the supernatant was filtered through a 0.45 μm pore membrane and stored at −20°C for SCFA quantification by gas chromatography. The pellets were stored at −80°C for the subsequent characterization of microbial populations.

### Determination of Organic Acids by Gas Chromatography

Chromatographic analysis was carried out using an Agilent 7890a GC system with a DB23 column (Agilent Technologies, Santa Clara, CA, United States) coupled to a flame detector (FID). The temperature at the injection port and at the FID was 250°C. Helium was used as carrier gas at a flow rate of 1.6 mL/min. For the run, 1 μL of the sample was injected with a 1:25 split and a temperature program that consisted of a ramp from 100 to 200°C at a speed of 8°C/min, keeping constant at 200°C for 3 min was used. Calibration curves with standards of glacial acetic acid (1–50 mM), propionic acid (0.5–20 mM), butyric acid (0.5–20 mM), iso-butyric acid (0.5–20 mM), and iso-valeric acid (0.5–20 mM) were prepared (Sigma-Aldrich, San Luis, MO, United States). Organic acids were identified by comparison to standard retention times and quantified with the corresponding peak area using the calibration curve. Differences were statistically tested using One-way analysis of variance (ANOVA) with Tukey’s multiple comparison test (*p* < 0.05) conducted by the GraphPad Prism^®^ software.

### DNA Isolation

DNA extractions were performed by using a commercial QIAamp PowerFecal DNA kit (Qiagen, Hilden, Germany) following the manufacturer’s instructions. DNA concentration and quality were determined using a NanoDrop spectrophotometer (Thermo Fisher Scientific, Waltham, MA, United States). The DNA samples were used for DGGE analysis and mass sequencing. DNA extracted from pure cultures of *Lactobacillus casei, L. plantarum, L. kefiri*, and *Bifidobacterium adolescentis* were used as reference strains for DGGE analysis.

### Microbiota Evolution Analysis by DGGE

The fecal microbiota evolution during batch fermentations was analyzed by partial amplification of the 16S rRNA gene using universal primers 518R and 338F-GC (Table 1). PCR amplification was performed using Taq polymerase Pegasus (PB-L Biological Products, Argentina) following the manufactures instruction and using 1 ng/μL of DNA template. The reaction was carried out in a T100 thermal cycler (Bio-rad laboratories, Irvine, CA, United States) with the following amplification program: 94°C for 5 min; 35 cycles of 94°C for 30 s, 60°C for 45 s and 72°C for 20 s; and a final extension step at 72°C for 1 min. The amplification products were analyzed by electrophoresis in 1% w/v agarose gels with ethidium bromide and revealed under UV light.

**TABLE 1:** Primers used in this study.

Primer name	Sequence	References
338f-GC	GCclamp-ACTCCTACGGGAGGCAGCAG	Bakke et al., 2011
518r	ATTACCGCGGCTGCTG	
515	GTGYCAGCMGCCGCGGTAA	Caporaso et al., 2011
806	GGACTACNVGGGTWTCTAAT	

Denaturing-gradient-gel electrophoresis (DGGE) was performed in a DGGE-2401 analyzer (C.B.S. Scientific Co., Del Mar, CA, United States). The PCR products (15 μL) were seeded in 8 g/100 mL polyacrylamide gels (15 × 20 × 0.075 cm) in TAE buffer [50X TAE is 2 M Tris, 1 M acetic acid, and 50 mM EDTA (pH 8.0)]. A denaturing gradient of Urea-Formamide 40–60% (100% corresponds to Urea 7 M and Formamide 40% v/v) was used to achieve the optimal separation of the bands corresponding to Eubacteria. Electrophoresis was carried out at 90 V for 16 h at 60°C. Gels were then stained by immersion for 30 min in a 0.1 μL/mL Sybr-Gold solution (Invitrogen, United States) in TAE buffer and observed under UV light. Band patterns obtained for each sample were compered using the Bionumerics 6 program (Applied maths NV, Sint-Martens-Latem, Belgium). The percentage of similarity between the samples was calculated using the Dice Similarity Coefficient and the corresponding UPGMA dendrograms were constructed.

### Mass Sequencing Analysis Using Ion Torrent PGM

The mass sequencing analysis was carried out in the MR DNA molecular research laboratory (TX, United States^1^), based on established and validated protocols^2^. Primers 515 and 806 that amplify the V4 variable region of the gene that codes for 16S rRNA were used (Table 1). For PCR, the HotStarTaq Plus Master Mix kit (Qiagen, Hilden, Germany) was used with an amplification program that consisted of 94°C 3 min, 30 cycles of 94°C 30 s, 53°C 40 s, 72°C 1 min and finally 72°C 5 min.

The mass sequencing analysis was performed using the Ion Torrent Personal Genome machine (PGM) system (Thermo Fisher Scientific, Waltham, MA, United States) following the manufacturer’s guidelines. The generated data was demultiplexed and analyzed using a pipeline developed in the MR DNA molecular research laboratory. Raw data sequencing reads were quality trimmed using the QIIME suite of tools. Sequences were depleted of barcodes and primers, followed by removal of short sequences (<150 bp), sequences with ambiguous base calls and with homopolymer runs exceeding 6 bp. Noise from sequences and chimeras were also removed. Sequencing data were grouped into 3% divergence operating taxonomic units (OTUs) and taxonomically classified using the BLASTn.NET algorithm with the database derived from RDPII^3^ and NCBI^4^.

## Results

### EPS Production by *L. paracasei* Strains in Milk

EPS production during milk fermentation at 30°C by *L. paracasei* CIDCA 8339 and CIDCA 83124 were about 130–145 and 140–160 mg of EPS per liter of fermented milk respectively. These values are within the expected range, since EPS yield by LAB is normally very low (Ruas-Madiedo et al., 2008; Llamas-Arriba et al., 2019). Crude EPS isolated from fermented milk with *L. paracasei* CIDCA 8339 (EPS_8339_) and CIDCA 83124 (EPS_83124_) were partially characterized by analyzing their molecular weight distribution (Mw) by gel permeation chromatography. EPS_8339_ consists of two fractions, a high Mw fraction of about 4 10^5^ Da and a low Mw fraction of about 1 10^4^ Da. On the other hand, EPS_83124_ presents four fractions: a low Mw fraction of 1 10^4^ Da, an intermediate Mw fraction of 7 10^4^ Da and a high Mw fraction constituted by two Mw distributions of 7 10^5^ and 6 10^6^ Da.

### Evaluation of Fecal Microbiota Evolution During EPS Fermentation

**FIGURE 1:**
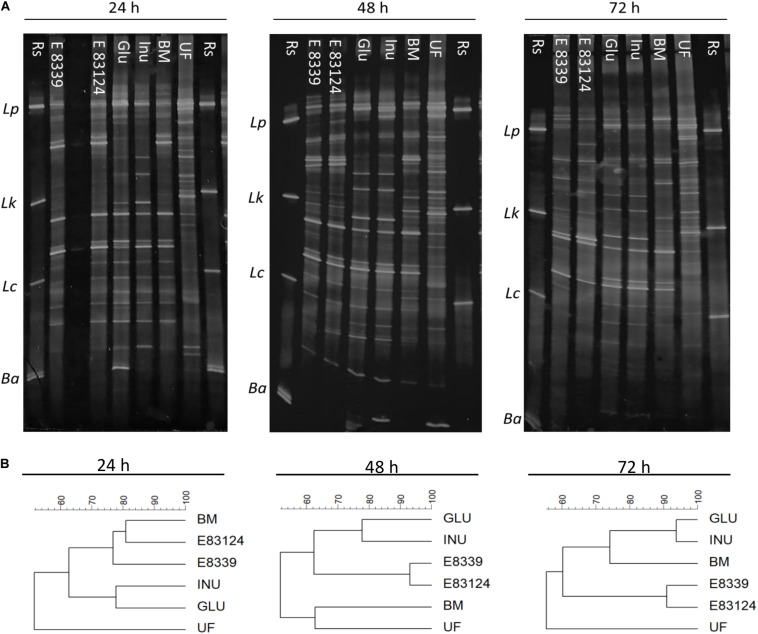
PCR-based DGGE profiles **(A)** and the respective dendrograms **(B)** of fecal homogenates after 24, 48, and 72 h fermentation with EPS_8339_ (E 8339), EPS_83124_ (E 83124), glucose (Glu), inulin (Inu) or without extra sugar added (BM). UF profile corresponds to the one obtained with the unfermented fecal homogenate and Rs corresponds to reference strains. Lp, *Lactobacillus. plantarum*; Lk, *Lactobacillus kefiri;* Lc, *Lactobacillus casei;* Ba, *Bifidobacterium adolescentis.* Dendrograms were obtained by similarity analysis of DGGE profiles using Dice similarity coefficient and UPGMA cluster analysis.

PCR DGGE profiles were employed to monitor major qualitative changes in the compositions of microbial groups of fecal homogenates with and without sugar source added after 24, 48, and 72 h fermentation. DGGE profiles obtained were both time and sugar source dependent (Figure 1A). After 24 h of incubation, DGGE profiles of the homogenates fermented in the presence of EPS_8339_ and EPS_83124_ had a high similarity percentage (77–81%) with respect to the profile obtained for those grown in the carbohydrate-free basal medium (Figure 1B). In contrast, profiles of homogenates fermented with glucose or inulin presented less similarity when compared to the basal medium. However, after 72 h fermentation, the electrophoretic profile of the homogenates fermented in the presence of glucose and inulin resembled more the profile obtained in the basal medium, while those fermented in the presence of EPS_8339_ and EPS_83124_ showed a different band pattern, locating in a separated cluster and showing only 60% similarity with the two controls (GLU and INU). In the presence of glucose or inulin, changes in microbiota were observed after short fermentation times indicating the rapid use or assimilation of these sugars by fecal microorganisms. On the other hand, when EPS_8339_ and EPS_83124_ were added to basal medium, the changes in fecal microbiota occurred after long fermentation times, probably because the microorganisms need to adapt to this new carbon source.

**FIGURE 2:**
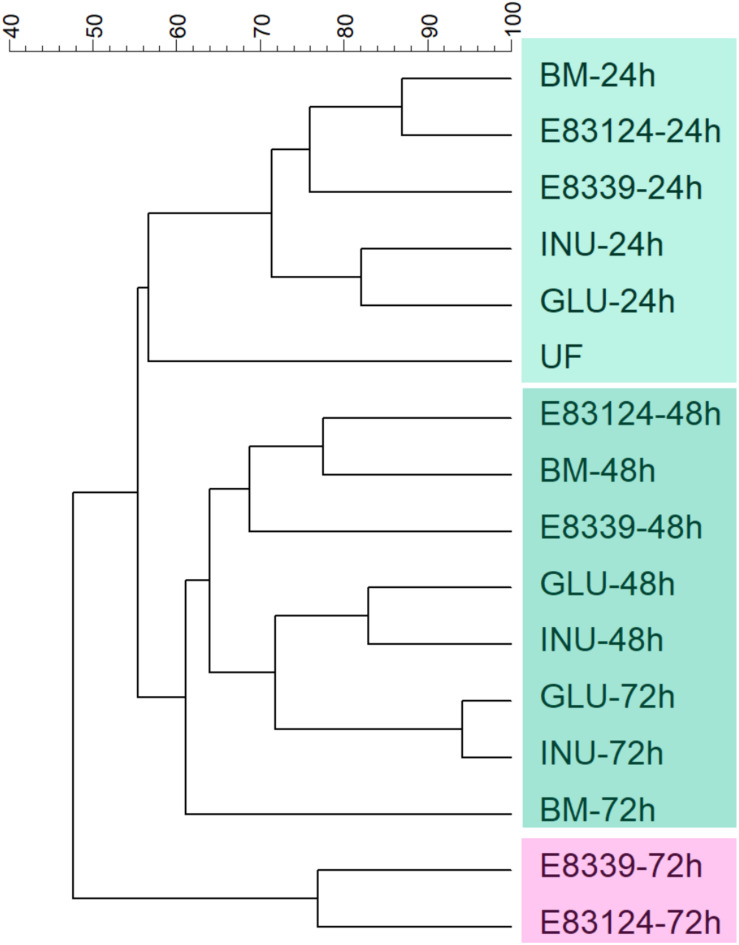
UPGMA dendrogram of DGGE profiles of fecal homogenates microbiota after different fermentation times with EPS_8339_ (E 8339), EPS_83124_ (E 83124), glucose (GLU), inulin (INU) or without sugar source added (BM). UF corresponds to the unfermented fecal homogenate. Dendrogram was obtained by similarity analysis of DGGE profiles using Dice similarity coefficient and UPGMA cluster analysis.

Dendrogram comparing V3 DGGE profiles obtained with all growth media assayed at different fermentation times is shown in Figure 2. Two main clusters that have a similarity value lower than 48% were observed. One of them joined the samples corresponding to the 72 h of fermentation in the media containing EPS_8339_ and EPS_83124_ and the other cluster joined the rest samples grouped in two subclusters. It can be observed that all the samples obtained after 24 h fermentation joined in the same subcluster with 71% similarity. The other subcluster grouped DGGE profiles of the rest of the samples obtained after 48 and 72 h fermentation (61% of similarity). Within this second subcluster inulin and glucose fermentation after 48 and 72 h grouped together with 72% of similarity.

Since fecal homogenates fermented during 72 h with EPS_8339_ and EPS_83124_ presented the higher differences in the DGGE band pattern compared to basal medium control, we proceed to perform a mass sequencing analysis of these samples using Ion Torrent PGM. Sequence depth performed in the analysis was adequate since rarefaction curves reached a plateau for all samples (Supplementary Figure S2). The analysis of alpha diversity across the samples gave Shannon diversity indices of 3.79, 3.23, and 3.30 for fermentation in carbohydrate-free basal medium, basal medium with EPS_8339_ and basal medium with EP_83124_, respectively, indicating high diversity in the three samples.

**FIGURE 3:**
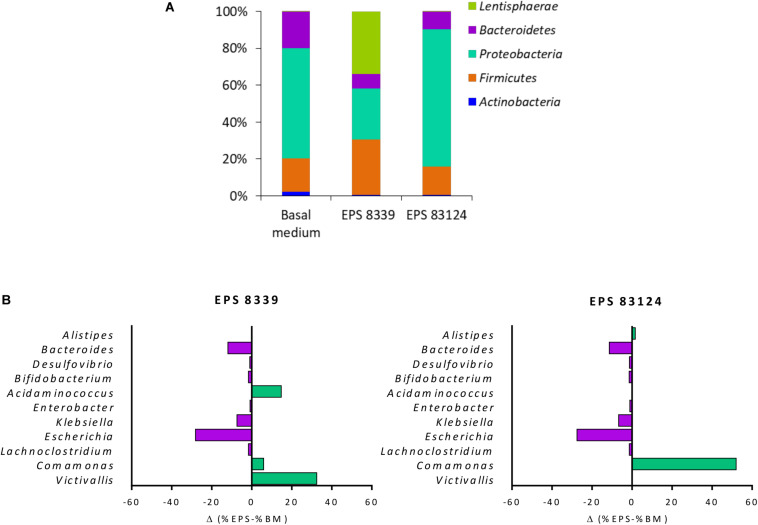
Relative abundance at the phylum level of fecal homogenates fermented for 72 h in the presence EPS_8339_, EPS_83124_ or without extra sugar added (BM). Only phyla with relative abundance higher than 1% in at least one sample were included **(A)**. Differences in bacterial relative abundance at the genus level in fecal homogenates fermented for 72 h with EPS_8339_ and EPS_83124_ in comparison with homogenates fermented 72 h without extra sugar added (BM). Only genera that showed differences higher than 1% in at least one sample were included **(B)**.

Figure 3A shows the distribution of the populations present in homogenates samples after 72 h fermentation at the phylum level. The main phyla present in the sample from basal medium (control) were *Proteobacteria* (58%), *Bacteroidetes* (19%), *Firmicutes* (17%), and *Actinobacteria* (2%). Fermentation of EPS_8339_ led to an increase in the relative proportion of *Firmicutes* (29%) and *Lentisphaerae* (32%), accompanied by the decrease in *Actinobacteria* (0.5%), *Proteobacteria* (27%) and *Bacteroidetes* (8%). On the other hand, fermentation of EPS_83124_ caused a reduction of the *Actinobacteria* (0.7%) and *Bacteroidetes* (9%) phyla with an increase in the proportion of *Proteobacteria* (73%).

At the genus level, it was observed that, regardless of the EPS used during the fermentation, the proportion of *Klebsiella* and *Escherichia* (γ*-Proteobacteria*) were reduced in 6 and 28% respectively while *Bacteroides* (*Bacteroidetes*) dropped in about 11–12% (Figure 3B). Nonetheless, the genera that showed an increment in their relative abundance after fermentation were different in both EPS samples, indicating that EPS_8339_ and EPS_83124_ are selectively used by different microorganisms present in fecal microbiota. The proportion of *Comamonas* (β*-Proteobacteria*) increased 6% for EPS_8339_ and 52% for EPS_83124_. This substantial rise of *Comamonas* genera explains the difference in the proportion of *Proteobacteria* phylum previously mentioned. Furthermore, fermentation of EPS_8339_ increased the proportion of the genera *Victivallis* (33%) and *Acidaminococcus* (15%) which correspond to almost the total rise in *Lentisphaerae* and *Firmicutes* phyla evidenced. However, fermentation of both EPS did not induce major changes in the population of *Lactobacillus* and *Bifidobacterium*.

### SCFA Production During EPS Fermentation by Fecal Microbiota in Batch Cultures

**FIGURE 4:**
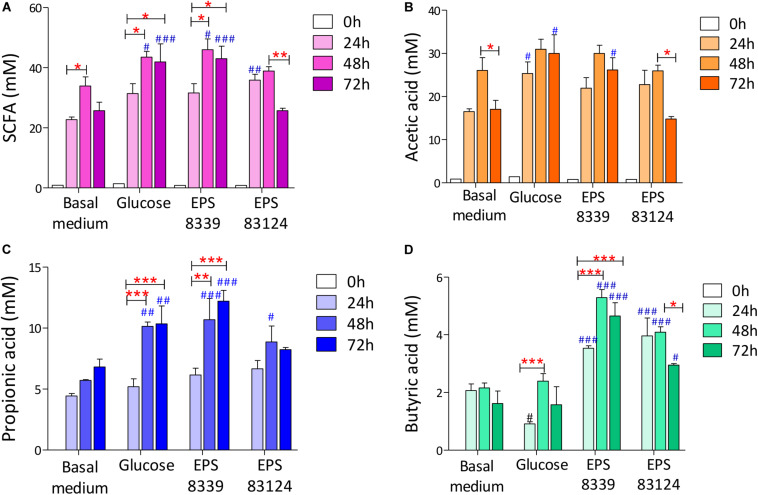
Total SCFA **(A)** and individual acetate **(B)**, propionate **(C)** and butyrate **(D)** levels of fecal homogenates fermented during 24, 48, and 72 h with and without sugar source added. # indicates significant differences against carbohydrate-free basal medium at the same fermentation time. *indicates significant differences between samples with a specific sugar at different fermentation time. (*/#*p* < 0.05, **/##*p* < 0.01, ***/###*p* < 0.001, Tukey’s multiple comparison test).

When analyzing the SCFA levels in the supernatant of fermented samples, it was evidenced that EPS_8339_ and EPS_83124_ are metabolized by the fecal microbiota producing, consequently, a significant increase in organic acids with recognized biological activity (propionate and butyrate) compared to basal medium. As an example, chromatograms obtained with fecal homogenates with EPS_83124_ after different fermentation times are shown in Supplementary Figure S3. Total SCFA levels increased during the first 24 and 48 h of incubation with and without external carbon sources while no changes or a decrease in total SCFA were observed after 72 h depending on the sugar source. Fermentation of EPS_8339_ showed a pattern similar to glucose, where the greatest increase in SCFA was evidenced at 48 and 72 h. The fermentation of EPS_83124_ showed a significant increase after 24 h followed by a drop after 72 h of fermentation. This reduction could be due to the fact that some SCFA produced can be then consumed by some microbial population whose activity was selectively stimulated by this EPS (Figure 4A).

On account of the levels of each individual SCFA with biological activity, it was observed that neither EPS_8339_ nor EPS_83124_ significantly increased acetate levels. However, in the case of EPS_83124_ a reduction in acetate levels was observed after 72 h fermentation similar to the results obtained with carbohydrate-free basal medium (Figure 4B). Regarding propionate, EPS_8339_ and glucose fermentation led to a significant increase of this organic acid at 48 and 72 h, while fermentation of EPS_83124_ produced a significant increase in propionate only at 48 h (Figure 4C). However, the levels of propionate achieved in the presence of EPS are low compared to inulin fermentation, since this prebiotic was metabolized by fecal microbiota producing propionate in concentrations of about 100 mM (Table 2), 10 times higher than those obtained with EPS_8339_, EPS_83124_ or glucose. Furthermore, it is noteworthy that fermentation of both EPS led to a significant increase in butyrate at all fermentation times, unlike the results observed with glucose and inulin where no increment in butyrate was observed (Figure 4D and Table 2).

**TABLE 2:** Short chain fatty acids (SCFA) concentration of the homogenates fermented in basal medium with inulin at different times.

	Fermentation time		
	
	24 h	48 h	72 h
Total SCFA (mM)	131.90 ± 0.67	150.25 ± 34.20	135.00 ± 12.51
Acetic acid (mM)	27.25 ± 1.33	31.41 ± 6.52	31.63 ± 2.91
Propionic acid (mM)	102.29 ± 0.41	116.21 ± 27.17	101.78 ± 9.55
Butyric acid (mM)	1.59 ± 0.21	2.63 ± 0.51	1.59 ± 0.17

**Significant differences (p < 0.05, Tukey’s multiple comparison test) were no detected at different fermentation times.**

These results show that EPS_8339_ fermentation led to an increment of propionate and butyrate, while fermentation of EPS_83124_ increased butyrate levels. Noteworthy, both EPS generated a different pattern of SCFA than inulin fermentation, which significantly increases propionate but does not modify butyrate levels.

In addition, two peaks that elute at 2.63 and 3.25 min and that were identified as isobutyric and isovaleric acid respectively, progressively increased with fermentation time when both bacterial EPS were used as sugar sources (Table 3). In contrast, these organic acids were not detected when inulin or glucose were present.

**TABLE 3:** Isobutyric and isovaleric levels from homogenates fermented in basal medium with or without EPS at different incubation times.

	Fermentation time (h)	Isobutyric acid (mM)	Isovaleric acid (mM)
Basal medium	24	0.37 ± 0.03 ^a^	0.24 ± 0.17 ^a^
	48	0.79 ± 0.13^ab^	0.45 ± 0.06^a^
	72	1.15 ± 0.50^b^	0.90 ± 0.55^a^
Basal medium with EPS_8339_	24	0.37 ± 0.12 ^a^	0.20 ± 0.13^a^
	48	1.28 ± 0.45 ^b^	2.86 ± 1.74 ^b^
	72	3.03 ± 0.20 ^c^	6.01 ± 0.65 ^c^
Basal medium with EPS_38124_	24	0.30 ± 0.09 ^a^	ND
	48	1.30 ± 0.51 ^b^	2.59 ± 1.26 ^b^
	72	2.27 ± 0.01 ^d^	4.00 ± 0.012 ^b^

**ND, non-detected. Different letters indicate significant differences (p < 0.05, Tukey’s multiple comparison test).**

## Discussion

The understanding of the mechanisms by which a balanced microbiome contributes to health has been considerably expanded in the last years, being the role of the metabolites one of the focus of research. In particular, SCFA results of great interest because of their wide variety of health benefit effects at both intestinal and extra-intestinal level. The homeostasis of the intestinal microbiota and its corresponding metabolome depends on the characteristics of the host (age, sex, genetic background) and on environmental conditions (stress, medications, gastrointestinal surgery, infectious, and toxic agents) (Conlon and Bird, 2015). Although microbiota composition is diverse between individuals in terms of genus and species, it is dominated by four main phyla: *Firmicutes, Bacteroidetes, Actinobacteria*, and *Proteobacteria* (Hollister et al., 2014; Hugon et al., 2015) as it was described in the present work. It is worth to note that the percentage of each phylum described herein was in concordance to those described previously (Milani et al., 2017). The early colonization process is crucial for long-term health benefits having the infant gut microbiota a main role in modulating risk factors related to adult health conditions (Milani et al., 2017). It was observed that gut bacterial microbiome rapidly diversifies over the first years of life in healthy children while is less diverse in those who develop allergy or asthma or who are malnourished (Durack and Lynch, 2019). As the composition of the infant’s microbiome can have a profound effect on adult life, the search of new compounds that contribute to microbiota modulation for their inclusion in infant diet results of relevance. In the present work, the changes in infant fecal microbiota and SCFA levels induced by EPS_8339_ and EPS_83124_ fermentation were studied. For that purpose, fecal homogenates prepared in a basal medium were added with different sugars including glucose and inulin that were used as controls.

Lactic acid bacteria exopolysaccharides are widely studied for its contribution to food texture. Still, they can also act as bioactive compounds that are able to exert their effect by direct interaction to the epithelial cells or indirectly by inducing specific changes in the composition and metabolic activity of the intestinal microbiota (Gibson et al., 2017). They are highly diverse in structure and sugar composition and it has been evidenced that the biological activity attributed to each biopolymer, such as the prebiotic potential, is mostly dependent on its molecular characteristics (Salazar et al., 2016). The fermentation of EPS synthetized by *L. paracasei* CIDCA 8339 and CIDCA 83124 led to changes in fecal microbiota as well as in SCFA profile. These results indicate in first place, that both EPS are fermentable by fecal microbiota. However, EPS synthetized by *L. paracasei* CIDCA 8339 and CIDCA 83124 in milk showed different molecular weight distribution. Thus, it is not surprising that the changes in the fecal microbiota and in SCFA profile exerted by both polymers are different.

When analyzing the mass sequencing data from 72 h fermentation samples, it was evidenced that neither EPS_8339_ nor EPS_83124_ favored the growth of the genera commonly consider as beneficial such as *Lactobacillus* and *Bifidobacterium*. Moreover, despite the production of propionate and butyrate evidenced in these samples, none of the microorganisms generally associated to the production of those SCFA including *Faecalibacterium prausnitzii, Eubacterium rectale, Eubacterium hallii, Ruminococcus bromii, Akkermansia municiphilla*, and *Roseburia intestinalis* (Morrison and Preston, 2016) were increased after 72 h fermentation when compared to basal medium. It has been reported that species from the genera *Victivallis, Acidaminococcus* and *Comamonas*, the most favored by EPS_8339_ and EPS_83124_ fermentation, are part of the human gastrointestinal tract (Jumas-Bilak et al., 2007; Segata et al., 2012; Samb-Ba et al., 2014; Ricaboni et al., 2017). However, they have not been widely studied and their role in the intestinal microbiota is not clearly yet. Although they do not correspond to any of the genera commonly associated with the production of SCFA, it has been reported in the literature that *Acidaminococcus* species are capable of producing acetate, propionate and butyrate (Jumas-Bilak et al., 2007). Furthermore, the genus *Victivallis* also contributes to acetate production (Zoetendal et al., 2003). Despite no significant increase in acetate levels were observed in the samples fermented in the presence of EPS, it must be considered that acetate can be used by many gut commensals to produce propionate and butyrate in a growth-promoting cross-feeding process (Verkhnyatskaya et al., 2019). Considering this, the production of acetate by *Acidaminococcus* and *Victivallis* could indirectly promote the production of other SCFA, such as butyrate through the butyryl-CoA: acetate-CoA transferase pathway present in some microorganisms of the microbiota (Louis and Flint, 2017). In this way, these populations could be directly or indirectly responsible for the significant increase in butyrate observed during fermentation of EPS_8339_. The fermentation of EPS_83124_ results in an increase of species of the genera *Comamonas* such as *C. aquatica* and *C. kerstersii* (data not shown) that do not produce SCFA but instead are able to consume them (Wauters et al., 2003). Therefore, the pronounced increase in *Comamonas* due to EPS_83124_ fermentation could explain the significant drop in acetate and butyrate evidenced after 72 h fermentation. It has been evidenced that the presence of this genus, that is normally located in the Lieberkühn crypts, is beneficial since it participates in the maintenance of local homeostasis that is essential for epithelial regeneration (Pédron et al., 2012). Similarly, the consumption of milk containing *L. casei* BL23 in Balb/c mice also generated a significant increase in *Comamonas* (Yin et al., 2014). Moreover, the significant decrease in the *Enterobacteriaceae* family, that include genera usually associated with pathogens (*Klebsiella* and *Escherichia*), evidenced with both EPS also contributes to a more anti-inflammatory and healthy gut state.

It can also be highlighted that results obtained with these EPS differ from the results obtained with inulin that was included as a positive prebiotic control. Fermentation of both EPS conducted to a different SCFA profile and led to greater changes in the microbial population after 72 h fermentation. In the DGGE profiles obtained for glucose (a sugar easily fermentable by microorganisms that was included as control of bacterial growth and activity) and inulin it can be observed a high percentage of similarity between both sugars at 48 and 72 h fermentation with practically identical band patterns. These similar patterns obtained with inulin and glucose could be attributed to the fact that the donors of fecal samples regularly consumed formula milk with inulin, FOS or GOS, so their microbiota is probably adapted to inulin-type prebiotics. These results highlight the relevance of finding new compounds that can exert a beneficial effect on the intestinal microbiota different from those observed with the prebiotics commonly used in food in order to contribute to establish a widely diverse intestinal microbiota usually associated with a healthy state (Durack and Lynch, 2019). In this context, the EPS produced by *L. paracasei* CIDCA 8339 and CIDCA 83124 emerge as alternative potential prebiotics that can be fermented by the fecal microbiota *in vitro*, producing modifications in the DGGE microbiological profile after 72 h that differs from the changes induced by inulin. Additionally, in contrast to inulin that induced mainly the production of propionate, the fermentation of EPS_8339_ and EPS_83124_ led to a significant increase in butyrate levels, a bioactive metabolite with several beneficial effects at the intestinal level. It is remarkable that, even though bacterial EPS_8339_ and EPS8_3124_ required 72 h fermentation to induce changes in microbial population, they rapidly modified microbiota activity as can be evidenced by the increase in SCFA after 24 h. Considering this, both EPS could be used as complementary prebiotics that contribute to consumers’ health inducing favorable changes at the intestinal microbiota that are different from the ones induced by inulin.

The increase of butyrate as a consequence of EPS_8339_ and EPS_83124_ fermentation may bring a wide range of health benefits, particularly at the intestinal level. Butyrate can be used as an energy source by enterocytes (van der Beek et al., 2015). Moreover, it contributes to strengthen the intestinal epithelial barrier through a mechanism that involves the induction of tight junctions’ proteins expression such as Claudin 1 and ZO-1 and their redistribution in the membrane (Morrison and Preston, 2016). The loss of integrity of the intestinal barrier and the consequent increase in its permeability is generally associated with an increase in bacterial translocation and/or its wall components, which results in a mild chronic inflammatory state that has been associated with pathologies such as obesity, insulin resistance and diabetes type 2 (Cani et al., 2008; Qin et al., 2012). Furthermore, propionate production would also be beneficial in people that suffer of obesity since it inhibits cholesterol synthesis at the liver, regulates lipogenesis in adipose tissue (Ríos-Covián et al., 2016; Tsai et al., 2019) and regulates appetite through the expression of leptin, PYY and GLP-1 (Chambers et al., 2015). Thus, the use of prebiotics that leads to the production of butyrate and/or propionate at the intestinal level results interesting in individuals with these kinds of metabolic disorders. On the other hand, SCFA regulate the immune response at the intestinal level, contributing to the healthy state in patients suffering from inflammatory bowel diseases. Butyrate, for instance, exerts an anti-inflammatory effect by inhibiting the activation of the transcription factor NFκB in macrophages and the expression of proinflammatory cytokines (IL-6 and IL-12) in dendritic cells. Moreover, butyrate and propionate are able to regulate the production and function of regulatory T cells by inhibiting histone deacetylases (Morrison and Preston, 2016; Requena et al., 2018). Dysbiosis observed in inflammatory bowel diseases, including ulcerative colitis and Crohn’s disease, is generally associated with a reduction in SCFA levels (Alagón Fernández del Campo et al., 2019) and an increase in species of the *Enterobacteriaceae* family and other opportunistic pathogens (Gonçalves et al., 2018; Uchiyama et al., 2019). The microbiota modulation favoring the production of SCFA during infancy is relevant to reduce the risk of disease development in the future. In this context, Roduit et al. (2019) studied the role of SCFA in the prevention of allergy and asthma by analyzing SCFA levels in 1-year-old children fecal samples and correlating them with the development of disease when those children were 6 years old. The authors evidenced that children that presented the highest levels of propionate and/or butyrate when they were 1 year old, were less likely to develop asthma, food allergy and allergic rhinitis when they grew up. In the same way, it has been suggested that butyrate-producing bacteria play a key role in reducing the risk of developing type 1 diabetes in children between 1 and 5 years old (de Goffau et al., 2014).

This study revealed that the EPS produced by *L. paracasei* CIDCA 8339 and CIDCA 83124 isolated from kefir induce substantial distinct effects on fecal microbiota activity and composition of healthy children leading to selective enrichments of those microorganisms that possess the ability to adapt their growth to the respective substrates. Mass sequencing analysis demonstrated that the fermentation of EPS_8339_ leads to an increase in the proportion of the genera *Victivallis*, *Acidaminococcus*, and *Comamonas* and a significant drop in the proportion of enterobacteria. In the same direction, the fermentation of the EPS_83124_ also resulted in a marked reduction in the population of Enterobacteriaceae with a significant increase in the genus *Comamonas.* These responses were linked to directed changes in SCFA toward butyrate production in higher concentration than controls. It was observed that both EPS presented a different fermentation profile probably due to differences in their structural characteristics. EPS_8339_ fermentation led to an increment of propionate and butyrate, while fermentation of EPS_83124_ increased mainly butyrate levels. These increase in the production of propionate and/or butyrate, accompanied by a decrease in the population of Enterobacteriaceae allowed us to hypothesize that the consumption of both EPS could contribute to reduce the inflammation at the intestinal level.

Although fecal microbiota composition partially correlates with gut microbiota, these results are a first step in the knowledge of the ability of two EPS from *L. paracasei* strains isolated from kefir to be fermented by human microbiota. It can be concluded that the EPS synthesized by *L. paracasei* CIDCA_8339_ and CIDCA_83124_ in milk can be considered bioactive compounds that modify the microbiota increasing the production of propionic and/or butyric acid, two metabolites highly associated with beneficial effects both at the gastrointestinal and extra-intestinal level.

## Data Availability Statement

SRA data generated in this study was uploaded in NCBI database. Project number PRJNA665182. BioSample accessions SAMN16245014, SAMN16245015, and SAMN16245016.

## Author Contributions

AB contributed in study design and conception, and performed experimental work, data interpretation, and manuscript writing. CD contributed with DGGE experiments, and contributed to microbiota data interpretation and writing the manuscript. NG contributed to acid organic determination and DNA extraction and revised the manuscript. GG participated in study design and conception, funding, and manuscript revising. AA participated in study design and conception, funding, and manuscript revising. All authors contributed to the article and approved the submitted version.

## Conflict of Interest

The authors declare that the research was conducted in the absence of any commercial or financial relationships that could be construed as a potential conflict of interest.

## Footnotes

1 www.mrdnalab.com, Shallowater,

2 http://www.mrdnalab.com/

3 http://rdp.cme.msu.edu

4 http://www.ncbi.nlm.nih.gov

## References

[B1] Aguirre M. Ramiro-Garcia J. Koenen M. E. Venema K. (2014). To pool or not to pool? Impact of the use of individual and pooled fecal samples for in vitro fermentation studies. *J. Microbiol. Methods* 107 1–7. 10.1016/j.mimet.2014.08.022 25194233

[B2] Alagón Fernández del Campo P. De Orta Pando A. Straface J. I. López Vega J. R. Toledo Plata D. Niezen Lugo S. F. (2019). The use of probiotic therapy to modulate the gut microbiota and dendritic cell responses in inflammatory bowel diseases. *Med. Sci.* 7 1–17. 10.3390/medsci7020033 30813381 PMC6410300

[B3] Bakke I. De Schryver P. Boon N. Vadstein O. (2011). PCR-based community structure studies of Bacteria associated with eukaryotic organisms: a simple PCR strategy to avoid co-amplification of eukaryotic DNA. *J. Microbiol. Methods* 84 349–351. 10.1016/j.mimet.2010.12.015 21182876

[B4] Balzaretti S. Taverniti V. Guglielmetti S. Fiore W. Minuzzo M. Ngo H. N. (2017). A novel rhamnose-rich hetero-exopolysaccharide isolated from *Lactobacillus paracasei* DG activates THP-1 human monocytic cells. *Appl. Environ. Microbiol.* 83 e2702–e2716. 10.1128/aem.02702-16 27913418 PMC5244303

[B5] Bengoa A. A. Iraporda C. Acurcio L. B. de Cicco Sandes S. H. Costa K. Moreira Guimarães G. (2019a). Physicochemical, immunomodulatory and safety aspects of milks fermented with *Lactobacillus paracasei* isolated from kefir. *Food Res. Int.* 123 48–55. 10.1016/j.foodres.2019.04.041 31284997

[B6] Bengoa A. A. Iraporda C. Garrote G. L. Abraham A. G. (2019b). Kefir micro-organisms: their role in grain assembly and health properties of fermented milk. *J. Appl. Microbiol.* 126 686–700. 10.1111/jam.14107 30218595

[B7] Bengoa A. A. Llamas M. G. Iraporda C. Dueñas M. T. Abraham A. G. Garrote G. L. (2018a). Impact of growth temperature on exopolysaccharide production and probiotic properties of *Lactobacillus paracasei* strains isolated from kefir grains. *Food Microbiol.* 69 212–218. 10.1016/j.fm.2017.08.012 28941904

[B8] Bengoa A. A. Zavala L. Carasi P. Trejo S. A. Bronsoms S. Serradell M. (2018b). Simulated gastrointestinal conditions increase adhesion ability of *Lactobacillus paracasei* strains isolated from kefir to Caco-2 cells and mucin. *Food Res. Int.* 103 462–467. 10.1016/j.foodres.2017.09.093 29389636

[B9] Bradford M. (1976). A rapid and sensitive method for the quantitation of microgram quantities of protein utilizing the principle of protein-dye binding. *Anal. Biochem.* 72 248–254. 10.1006/abio.1976.9999 942051

[B10] Braegger C. Chmielewska A. Decsi T. Kolacek S. Mihatsch W. Moreno L. (2011). Supplementation of infant formula with probiotics and/or prebiotics: a systematic review and comment by the ESPGHAN committee on nutrition. *J. Pediatr. Gastroenterol. Nutr.* 52 238–250. 10.1097/MPG.0b013e3181fb9e80 21150647

[B11] Cani P. D. (2018). Human gut microbiome: hopes, threats and promises. *Gut* 67 1716–1725. 10.1136/gutjnl-2018-316723 29934437 PMC6109275

[B12] Cani P. D. Bibiloni R. Knauf C. Neyrinck A. M. Delzenne N. M. (2008). Changes in gut microbiota control metabolic diet-induced obesity and diabetes in mice. *Diabetes Metab. Res. Rev.* 57 1470–1481. 10.2337/db07-1403 18305141

[B13] Caporaso J. G. Lauber C. L. Walters W. A. Berg-Lyons D. Lozupone C. A. Turnbaugh P. J. (2011). Global patterns of 16S rRNA diversity at a depth of millions of sequences per sample. *Proc. Natl. Acad. Sci. U.S.A.* 108 4516–4522. 10.1073/pnas.1000080107 20534432 PMC3063599

[B14] Chambers E. S. Viardot A. Psichas A. Morrison D. J. Murphy K. G. Zac-Varghese S. E. K. (2015). Effects of targeted delivery of propionate to the human colon on appetite regulation, body weight maintenance and adiposity in overweight adults. *Gut* 64 1744–1754. 10.1136/gutjnl-2014-307913 25500202 PMC4680171

[B15] Conlon M. Bird A. (2015). The impact of diet and lifestyle on gut microbiota and human health. *Nutrients* 7 17–44. 10.3390/nu7010017 25545101 PMC4303825

[B16] de Goffau M. C. Fuentes S. van den Bogert B. Honkanen H. de Vos W. M. Welling G. W. (2014). Aberrant gut microbiota composition at the onset of type 1 diabetes in young children. *Diabetologia* 57 1569–1577. 10.1007/s00125-014-3274-0 24930037

[B17] Durack J. Lynch S. V. (2019). The gut microbiome: relationships with disease and opportunities for therapy. *J. Exp. Med.* 216 20–40. 10.1084/jem.20180448 30322864 PMC6314516

[B18] Ewaschuk J. B. Dieleman L. A. (2006). Probiotics and prebiotics in chronic inflammatory bowel diseases. *World J. Gastroenterol.* 12 5941–5950. 10.3748/wjg.v12.i37.5941 17009391 PMC4124400

[B19] Gareau M. G. Sherman P. M. Walker W. A. (2010). Probiotics and the gut microbiota in intestinal health and disease. *Nat. Rev. Gastroenterol. Hepatol.* 7 503–514. 10.1038/nrgastro.2010.117 20664519 PMC4748966

[B20] Garrote G. L. Abraham A. G. De Antoni G. L. (2010). “Microbial interactions in kefir: a natural probiotic drink,” in *Biotechnology of Lactic Acid Bacteria*, eds Mozzi F. Raya R. R. Vignolo G. M. (Ames, IO: Wiley-Blackwell), 327–340.

[B21] Gibson G. R. Hutkins R. Sanders M. E. Prescott S. L. Reimer R. A. Salminen S. J. (2017). Expert consensus document: the international scientific association for probiotics and prebiotics (ISAPP) consensus statement on the definition and scope of prebiotics. *Nat. Rev. Gastroenterol. Hepatol.* 14 491–502. 10.1038/nrgastro.2017.75 28611480

[B22] Gonçalves P. Araújo J. R. Di Santo J. P. (2018). A cross-talk between microbiota-derived short-chain fatty acids and the host mucosal immune system regulates intestinal homeostasis and inflammatory bowel disease. *Inflamm. Bowel Dis.* 24 558–572. 10.1093/ibd/izx029 29462379

[B23] Hamet M. F. Londero A. Medrano M. Vercammen E. Van Hoorde K. Garrote G. L. (2013). Application of culture-dependent and culture-independent methods for the identification of *Lactobacillus kefiranofaciens* in microbial consortia present in kefir grains. *Food Microbiol.* 36 327–334. 10.1016/j.fm.2013.06.022 24010614

[B24] Hamet M. F. Medrano M. Pérez P. F. Abraham A. G. (2016). Oral administration of kefiran exerts a bifidogenic effect on BALB/c mice intestinal microbiota. *Benef. Microb.* 7 237–246. 10.3920/BM2015.0103 26689227

[B25] Hamet M. F. Piermaria J. A. Abraham A. G. (2015). Selection of EPS-producing *Lactobacillus strains* isolated from kefir grains and rheological characterization of the fermented milks. *LWT Food Sci. Technol.* 63 129–135. 10.1016/j.lwt.2015.03.097

[B26] Hollister E. B. Gao C. Versalovic J. (2014). Compositional and functional features of the gastrointestinal microbiome and their effects on human health. *Gastroenterology* 146 1449–1458. 10.1053/j.gastro.2014.01.052 24486050 PMC4181834

[B27] Hugon P. Dufour J.-C. Colson P. Fournier P.-E. Sallah K. Raoult D. (2015). A comprehensive repertoire of prokaryotic species identifi ed in human beings. *Lancet Infect. Dis.* 15 1211–1230. 10.1016/S1473-3099(15)00293-5 26311042

[B28] Jumas-Bilak E. Carlier J. P. Jean-Pierre H. Mory F. Teyssier C. Gay B. (2007). *Acidaminococcus intestini* sp. nov., isolated from human clinical samples. *Int. J. Syst. Evol. Microbiol.* 57 2314–2319. 10.1099/ijs.0.64883-0 17911303

[B29] Kasubuchi M. Hasegawa S. Hiramatsu T. Ichimura A. Kimura I. (2015). Dietary gut microbial metabolites, short-chain fatty acids, and host metabolic regulation. *Nutrients* 7 2839–2849. 10.3390/nu7042839 25875123 PMC4425176

[B30] Koh A. De Vadder F. Kovatcheva-Datchary P. Bäckhed F. (2016). From dietary fiber to host physiology: short-chain fatty acids as key bacterial metabolites. *Cell* 165 1332–1345. 10.1016/j.cell.2016.05.041 27259147

[B31] Llamas-Arriba M. G. Hernández-Alcántara A. M. Yépez A. Aznar R. Dueñas M. T. López P. (2019). Functional and nutritious beverages produced by lactic acid bacteria. *Nutr. Beverag.* 12 419–465. 10.1016/b978-0-12-816842-4.00012-5

[B32] Louis P. Flint H. J. (2009). Diversity, metabolism and microbial ecology of butyrate-producing bacteria from the human large intestine. *FEMS Microbiol. Lett.* 294 1–8. 10.1111/j.1574-6968.2009.01514.x 19222573

[B33] Louis P. Flint H. J. (2017). Formation of propionate and butyrate by the human colonic microbiota. *Environ. Microbiol.* 19 29–41. 10.1111/1462-2920.13589 27928878

[B34] Louis P. Hold G. L. Flint H. J. (2014). The gut microbiota, bacterial metabolites and colorectal cancer. *Nat. Rev. Microbiol.* 12 661–672. 10.1038/nrmicro3344 25198138

[B35] Lynch K. M. Zannini E. Coffey A. Arendt E. K. (2018). Lactic acid bacteria exopolysaccharides in foods and beverages: isolation, properties, characterization, and health benefits. *Annu. Rev. Food Sci. Technol.* 9 155–176. 29580141 10.1146/annurev-food-030117-012537

[B36] Milani C. Duranti S. Bottacini F. Casey E. Turroni F. Mahony J. (2017). The first microbial colonizers of the human gut: composition, activities, and health implications of the infant gut microbiota. *Microbiol. Mol. Biol. Rev.* 81:e0036-17. 10.1128/mmbr.00036-17 29118049 PMC5706746

[B37] Miqdady M. Al Mistarihi J. Azaz A. Rawat D. (2020). Prebiotics in the infant microbiome: the past, present, and future. *Pediatr. Gastroenterol. Hepatol. Nutr.* 23 1–14. 10.5223/pghn.2020.23.1.1 31988871 PMC6966216

[B38] Morrison D. J. Preston T. (2016). Formation of short chain fatty acids by the gut microbiota and their impact on human metabolism. *Gut Microb.* 7 189–200. 10.1080/19490976.2015.1134082 26963409 PMC4939913

[B39] Offermanns S. (2014). Free fatty acid (FFA) and hydroxy carboxylic acid (HCA) receptors. *Annu. Rev. Pharmacol. Toxicol.* 54 407–434. 10.1146/annurev-pharmtox-011613-135945 24160702

[B40] Parker A. Fonseca S. Carding S. R. (2020). Gut microbes and metabolites as modulators of blood-brain barrier integrity and brain health. *Gut Microb.* 11 135–157. 10.1080/19490976.2019.1638722 31368397 PMC7053956

[B41] Pédron T. Mulet C. Dauga C. Frangeul L. Chervaux C. Grompone G. (2012). A crypt-specific core microbiota resides in the mouse colon. *mBio* 3:e00116-12. 10.1128/mBio.00116-12 22617141 PMC3372965

[B42] Piermaria J. A. de la Canal M. L. Abraham A. G. (2008). Gelling properties of kefiran, a food-grade polysaccharide obtained from kefir grain. *Food Hydrocoll.* 22 1520–1527. 10.1016/j.foodhyd.2007.10.005

[B43] Plé C. Breton J. Daniel C. Foligné B. (2015). Maintaining gut ecosystems for health: are transitory food bugs stowaways or part of the crew? *Int. J. Food Microbiol.* 213 139–143. 10.1016/j.ijfoodmicro.2015.03.015 25816749

[B44] Qin J. Li Y. Cai Z. Li S. Zhu J. Zhang F. (2012). A metagenome-wide association study of gut microbiota in type 2 diabetes. *Nature* 490 55–60. 10.1038/nature11450 23023125

[B45] Requena T. Martínez-Cuesta M. C. Peláez C. (2018). Diet and microbiota linked in health and disease. *Food Funct.* 9 688–704. 29410981 10.1039/c7fo01820g

[B46] Rezac S. Kok C. R. Heermann M. Hutkins R. (2018). Fermented foods as a dietary source of live organisms. *Front. Microbiol.* 9:1785. 10.3389/fmicb.2018.01785 30197628 PMC6117398

[B47] Ricaboni D. Mailhe M. Benezech A. Cadoret F. Fournier P. E. Raoult D. (2017). ‘*Acidaminococcus timonensis*’ sp. nov. and ‘*Acidaminococcus massiliensis*’ sp. nov. isolated from human gut. *New Microb. New Infect.* 15 46–48. 10.1016/j.nmni.2016.11.010 28018604 PMC5175988

[B48] Rimada P. S. Abraham A. G. (2003). Comparative study of different methodologies to determine the exopolysaccharide produced by kefir grains in milk and whey. *Lait* 83 79–87. 10.1051/lait:2002051

[B49] Ríos-Covián D. Ruas-Madiedo P. Margolles A. Gueimonde M. De los Reyes-Gavilán C. G. Salazar N. (2016). Intestinal short chain fatty acids and their link with diet and human health. *Front. Microbiol.* 7:185. 10.3389/fmicb.2016.00185 26925050 PMC4756104

[B50] Roberfroid M. Gibson G. R. Hoyles L. McCartney A. L. Rastall R. Rowland I. (2010). Prebiotic effects: metabolic and health benefits. *Br. J. Nutr.* 104 S1–S63. 10.1017/S0007114510003363 20920376

[B51] Roduit C. Frei R. Ferstl R. Loeliger S. Westermann P. Rhyner C. (2019). High levels of butyrate and propionate in early life are associated with protection against atopy. *Allergy* 74 799–809. 10.1111/all.13660 30390309

[B52] Round J. L. Mazmanian S. K. (2009). The gut microbiota shapes intestinal immune responses during health and disease. *Nat. Rev. Immunol.* 9 313–323. 10.1038/nri2515 19343057 PMC4095778

[B53] Ruas-Madiedo P. Abraham A. G. Mozzi F. de los Reyes-Gavilán C. G. (2008). “Functionality of exopolysaccharides produced by lactic acid bacteria,” in *Molecular Aspects of Lactic Acid Bacteria for Traditional and New Applications*, eds Mayo B. López P. Pérez-Martínez G. (Kerala: Research Signpost), 137–166.

[B54] Ryan P. M. Ross R. P. Fitzgerald G. F. Caplice N. M. Stanton C. (2015). Sugar-coated: Exopolysaccharide producing lactic acid bacteria for food and human health applications. *Food Funct.* 6 679–693. 10.1039/c4fo00529e 25580594

[B55] Salazar N. Binetti A. Gueimonde M. Alonso A. Garrido P. González del Rey C. (2011). Safety and intestinal microbiota modulation by the exopolysaccharide-producing strains *Bifidobacterium animalis* IPLA R1 and *Bifidobacterium longum* IPLA E44 orally administered to Wistar rats. *Int. J. Food Microbiol.* 144 342–351. 10.1016/j.ijfoodmicro.2010.10.016 21078530

[B56] Salazar N. Gueimonde M. Gonzalez de los Reyes-Gavilán C. Ruas-Madiedo P. (2016). Exopolysaccharides produced by lactic acid bacteria and *Bifidobacteria* as fermentable substrates by the intestinal microbiota. *Crit. Rev. Food Sci. Nutr.* 56 1440–1453. 10.1080/10408398.2013.770728 25675369

[B57] Salazar N. Gueimonde M. Hernández-Barranco A. M. Ruas-Madiedo P. Gonzalez de los Reyes-Gavilán C. (2008). Exopolysaccharides produced by intestinal *Bifidobacterium* strains act as fermentable substrates for human intestinal bacteria. *Appl. Environ. Microbiol.* 74 4737–4745. 10.1128/AEM.00325-08 18539803 PMC2519331

[B58] Samb-Ba B. Mazenot C. Gassama-Sow A. Gory Dubourg G. Richet H. Hugon P. (2014). MALDI-TOF identification of the human gut microbiome in people with and without diarrhea in senegal. *PLoS One* 9:e87419. 10.1371/journal.pone.0087419 24784934 PMC4006720

[B59] Segata N. Haake S. K. Mannon P. Lemon K. P. Waldron L. Gevers D. (2012). Composition of the adult digestive tract bacterial microbiome based on seven mouth surfaces, tonsils, throat and stool samples. *Genome Biol.* 13 1–18. 10.1186/gb-2012-13-6-r42 22698087 PMC3446314

[B60] Shen S. Wong C. H. (2016). Bugging inflammation: role of the gut microbiota. *Clin. Transl. Immunol.* 5:e72. 10.1038/cti.2016.12 27195115 PMC4855262

[B61] Thorburn A. N. Macia L. Mackay C. R. (2014). Diet, metabolites, and “Western-Lifestyle” inflammatory diseases. *Immunity* 40 833–842. 10.1016/j.immuni.2014.05.014 24950203

[B62] Thursby E. Juge N. (2017). Introduction to the human gut microbiota. *Biochem. J.* 474 1823–1836. 10.1042/BCJ20160510 28512250 PMC5433529

[B63] Tsai Y.-L. Lin T.-L. Chang C.-J. Wu T.-R. Lai W.-F. Lu C.-C. (2019). Probiotics, prebiotics and amelioration of diseases. *J. Biomed. Sci.* 26:3. 10.1186/s12929-018-0493-6 30609922 PMC6320572

[B64] Uchiyama K. Naito Y. Takagi T. (2019). Intestinal microbiome as a novel therapeutic target for local and systemic inflammation. *Pharmacol. Ther.* 199 164–172. 10.1016/j.pharmthera.2019.03.006 30877020

[B65] van der Beek C. M. Bloemen J. G. van den Broek M. A. Lenaerts K. Venema K. Buurman W. A. (2015). Hepatic uptake of rectally administered butyrate prevents an increase in systemic butyrate concentrations in humans. *J. Nutr.* 145 2019–2024. 10.3945/jn.115.211193 26156796

[B66] Venema K. Verhoeven J. Verbruggen S. Keller D. (2020). Xylo-oligosaccharides from sugarcane show prebiotic potential in a dynamic computer-controlled in vitro model of the adult human large intestine. *Benef. Microb.* 11 191–200. 10.3920/BM2019.0159 32208927

[B67] Verkhnyatskaya S. Ferrari M. De Vos P. Walvoort M. T. C. (2019). Shaping the infant microbiome with non-digestible carbohydrates. *Front. Microbiol.* 10:343. 10.3389/fmicb.2019.00343 30858844 PMC6397869

[B68] Vinolo M. A. R. Rodrigues H. G. Hatanaka E. Sato F. T. Sampaio S. C. Curi R. (2011). Suppressive effect of short-chain fatty acids on production of proinflammatory mediators by neutrophils. *J. Nutr. Biochem.* 22 849–855. 10.1016/j.jnutbio.2010.07.009 21167700

[B69] Wauters G. De Baere T. Willems A. Falsen E. Vaneechoutte M. (2003). Description of *Comamonas aquatica* comb. nov. and *Comamonas kerstersii* sp. nov. for two subgroups of *Comamonas terrigena* and emended description of *Comamonas terrigena*. *Int. J. Syst. Evol. Microbiol.* 53 859–862. 10.1099/ijs.0.02450-0 12807213

[B70] Weiss G. A. Hennet T. (2017). Mechanisms and consequences of intestinal dysbiosis. *Cell. Mol. Life Sci.* 74 2959–2977. 10.1007/s00018-017-2509-x 28352996 PMC11107543

[B71] Yin X. Yan Y. Kim E. B. Lee B. Marco M. L. (2014). Short communication: effect of milk and milk containing *Lactobacillus casei* on the intestinal microbiota of mice. *J. Dairy Sci.* 97 2049–2055. 10.3168/jds.2013-7477 24508432

[B72] Zavala L. Golowczyc M. A. Van Hoorde K. Medrano M. Huys G. Vandamme P. (2016). Selected *Lactobacillus strains* isolated from sugary and milk kefir reduce *Salmonella* infection of epithelial cells in vitro. *Benef. Microb.* 7 585–595. 10.3920/BM2015.0196 27291404

[B73] Zoetendal E. G. Plugge C. M. Akkermans A. D. de Vos W. M. (2003). *Victivallis vadensis* gen. nov., sp. nov., a sugar-fermenting anaerobe from human faeces. *Int. J. Syst. Evol. Microbiol.* 53 211–215. 12656175 10.1099/ijs.0.02362-0

