# Supplementary material for: Exopolysaccharides From Lactobacillus paracasei Isolated From Kefir as Potential Bioactive Compounds for Microbiota Modulation
Source: Front Microbiol. 2020 Oct 16;11:583254. doi: 10.3389/fmicb.2020.583254 (PMC7596202; doi:10.3389/fmicb.2020.583254)
Supplement: Supplementary file 1 [file Data_Sheet_1.pdf]

Name and surname :			
Age:			
Your son/daughter was born by:	Delivery	Caesarean section	
Did he/she have any hospitalization last year?	YES	NO	
Does he/she suffer a chronic disease?	YES	NO	Which one?
Does he/she take any medication?	YES	NO	Which one?
Did he/she consume antibiotics in the last 6 months?	YES	NO	
Breastfeeding?	YES	NO	For how long?
Does he/she consume formula milk?	YES	NO	Which one?
			Since when?
Indicate with a tick what kind of food does your kid consume			Vegetables
			Fruits
			Meat and poultry
			Fish
			Dairy products
			Grains,_Beans and Nuts
			Eggs

Supplementary Figure 1. Questioner answered by faecal samples donors' parents

A B

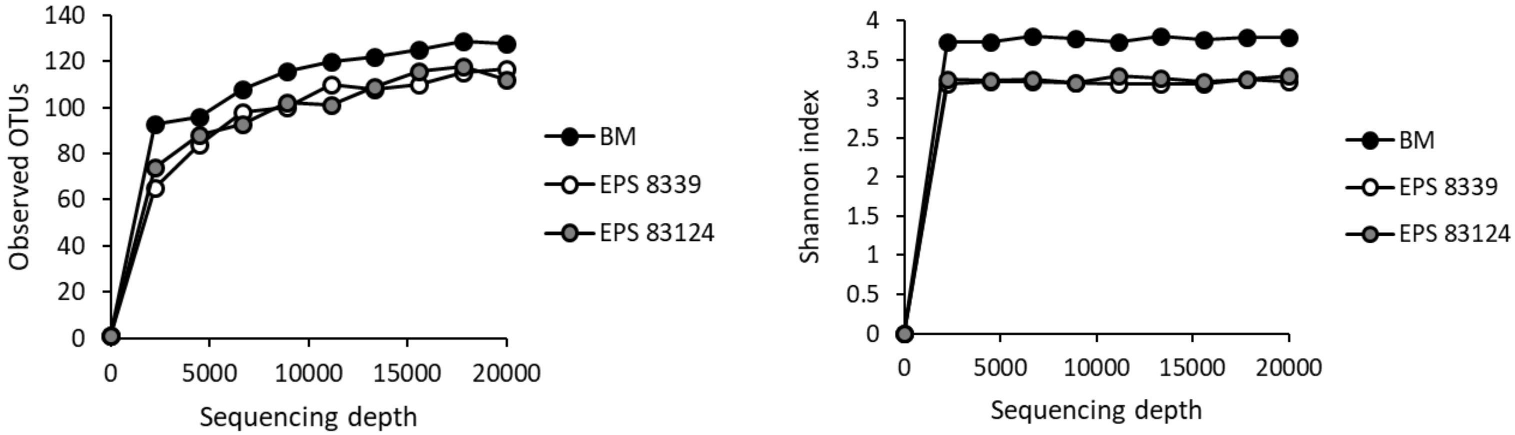

Supplementary Figure 2. Alpha-rarefaction curves of 16S RNA obtained from feacal homogenates after 72 h of fermentation on Basal Medium (BM), supplemented with EPS_{8339} or EPS_{83124} **A.** Observed OTUs. **B.** Shannon index.

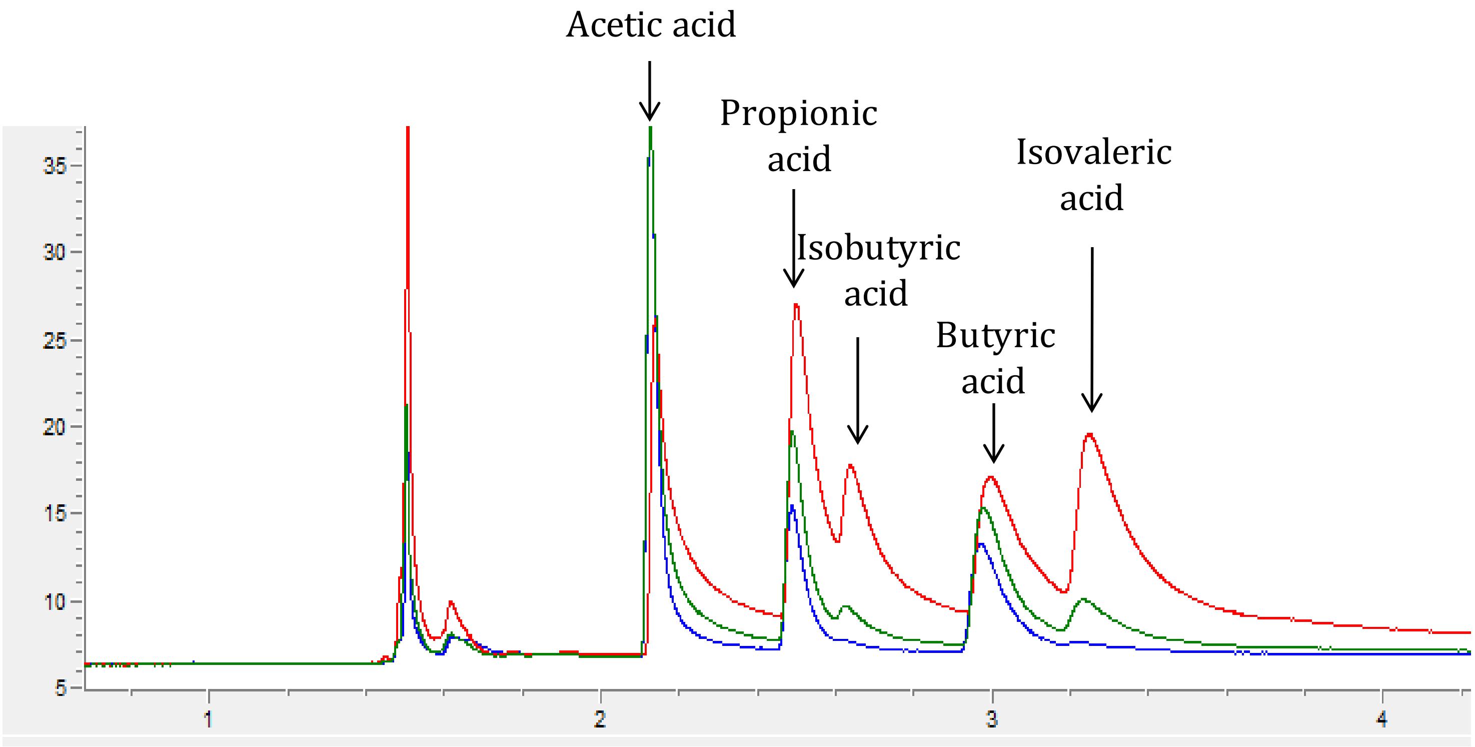

Supplementary Figure 3. Short chain fatty acids profile of faecal homogenates fermented with EPS_{83124} during 24 h (—), 48 h (—) and 72 h (—) determined by gas chromatography .
